# Phylo: A Citizen Science Approach for Improving Multiple Sequence Alignment

**DOI:** 10.1371/journal.pone.0031362

**Published:** 2012-03-07

**Authors:** Alexander Kawrykow, Gary Roumanis, Alfred Kam, Daniel Kwak, Clarence Leung, Chu Wu, Eleyine Zarour, Phylo players, Luis Sarmenta, Mathieu Blanchette, Jérôme Waldispühl

**Affiliations:** 1 School of Computer Science and McGill Centre for Bioinformatics, McGill University, Montreal, Quebec, Canada; 2 Nokia Research Center, Palo Alto, California, United States of America; Virginia Tech, United States of America

## Abstract

**Background:**

Comparative genomics, or the study of the relationships of genome structure and function across different species, offers a powerful tool for studying evolution, annotating genomes, and understanding the causes of various genetic disorders. However, aligning multiple sequences of DNA, an essential intermediate step for most types of analyses, is a difficult computational task. In parallel, citizen science, an approach that takes advantage of the fact that the human brain is exquisitely tuned to solving specific types of problems, is becoming increasingly popular. There, instances of hard computational problems are dispatched to a crowd of non-expert human game players and solutions are sent back to a central server.

**Methodology/Principal Findings:**

We introduce Phylo, a human-based computing framework applying “crowd sourcing” techniques to solve the Multiple Sequence Alignment (MSA) problem. The key idea of Phylo is to convert the MSA problem into a casual game that can be played by ordinary web users with a minimal prior knowledge of the biological context. We applied this strategy to improve the alignment of the promoters of disease-related genes from up to 44 vertebrate species. Since the launch in November 2010, we received more than 350,000 solutions submitted from more than 12,000 registered users. Our results show that solutions submitted contributed to improving the accuracy of up to 70% of the alignment blocks considered.

**Conclusions/Significance:**

We demonstrate that, combined with classical algorithms, crowd computing techniques can be successfully used to help improving the accuracy of MSA. More importantly, we show that an NP-hard computational problem can be embedded in casual game that can be easily played by people without significant scientific training. This suggests that citizen science approaches can be used to exploit the billions of “human-brain peta-flops” of computation that are spent every day playing games. Phylo is available at: http://phylo.cs.mcgill.ca.

## Introduction

The problem of optimally aligning a set of biological sequences (multiple sequence alignment (MSA)) is one of the most fundamental question in computational biology, with the first problem formulations and accompanying algorithms dating back to the early 1970's [1]. The goal of sequence alignment is to reveal sequence similarity by aligning together nucleotides (or amino acids) derived from a common ancestor or having an analogous role. Multiple alignments are at the core of most comparative genomics studies, as they allow to study how genetic sequences evolve and infer the function of different regions based on their evolutionary patterns [2], [3], including protein-coding regions [4] and RNA genes [5], as well as regulatory regions [6]–[8]. They also play a central role in the identification of genomic regions under purifying [9] or diversifying selection [10], [11]. Finally, they are essential for the prediction of the phenotypic impact of mutations in coding [12] or non-coding [13] regions.

Most mathematical formulations of MSA aim at identifying a maximum-scoring alignment, given a set of sequences. Although the sum-of-pairs score (which is defined as the sum, over all pairs of species, of the scores of the pairwise alignments induced by the MSA) has been heavily used in early studies, more phylogenetically-aware scoring schemes are now preferred [14]–[17]. Those approaches seek to identify a MSA, together with a set of ancestral sequences associated to the internal nodes of a given phylogenetic tree, that maximize the likelihood of the given set of sequences, under a given model of evolution. The MSA problem is NP-hard for all reasonable scoring schemes [18], and even the evaluation of the score of a given MSA is often also hard [19]. However, a large number of fast heuristics have been developed to align groups of DNA, RNA, and protein sequences (see [2], [3] for reviews). Despite the massive research efforts aiming at solving the MSA problem and its variations, this problem remains an active area of research, with important efforts in developing faster and more accurate algorithms for whole-genome MSA [16], [17], [20], [21], among others. Because of the sheer size of the sequences to be aligned (billions of nucleotides, in the case of mammalian genomes), a number of heuristics are required, often resulting in inaccuracies in the alignments produced. These inaccuracies have been shown to limit the accuracy of many of downstream analyses and it is thus of interest to reduce them.

To produce accurate alignments using a classical computational framework, exact and computationally intensive algorithms are required. Unfortunately, their usage on genome-scale problems clearly exceeds the capacity of even the most powerful computer clusters. In recent years, outsourcing has become a common strategy to address these computational limitations. The connection of thousands of individual computers through the internet network enabled to build giant virtual clusters with unmatched computing power, at a minimal cost. In 1999, the SETI@home project [22] pioneered this approach and demonstrated its efficiency. One year later, Vijay Pande and co-workers introduced this concept in the computational molecular biology research area and released the popular Folding@home program [23]. Nonetheless, even at a large scale, distributed computing remains limited by the algorithmic complexity of the method employed. For example, even small instances of problems such as the MSA's cannot be solved with guaranteed optimality in a reasonable time on single personal computers. A conceptual breakthrough is thus needed.

In classical outsourcing methods such as SETI@home and Folding@home, the bottleneck is twofold. First, the objective function can be hard to formalize. For instance, this is the case when the goal is to identify objects inside images. Next, even when fully defined, the objective function may not allow an efficient computing schema and thus require an exhaustive enumeration of the solution landscape. When the number of candidate solutions grows exponentially with the size of the input, this leads to computationally prohibitive algorithms. It turns out that these features characterize many real world problems. Interestingly, the human brain developed capabilities to efficiently address some of these problems. In particular, humans excel at visual pattern recognition. In such cases, the assistance of humans appears to be a reasonable option. This observation motivated the development of methods for harnessing these human abilities and has been embedded in a concept called *citizen science* [24].

Historically, the first attempt to apply citizen science principles was made by the Audubon Society's Christmas Bird Count, which started in 1900. However, the emergence of computers and of the internet greatly expanded the range of applications and the potential of this approach. Indeed, by developing human-computer interfaces that enable users to assist a computer program to solve a problem, and distributing this interface through the web, we can easily gather a large community of volunteers to help solving a given problem. In 2006, Stardust@home [25], followed one year later by Galaxy Zoo [26], pioneered these new research techniques. In the latter the users are asked to identify interstellar dust impacts or galaxies in pictures provided by a server. In 2008, Fold it [27] introduced these concepts in the field of molecular biology, focussing on the problem of proteins folding. Recent results suggest that for certain folding problems, solutions found by players were superior to those found by computers [28].

In this paper, we introduce Phylo, a citizen science framework to solve MSA problems. More specifically, Phylo aims to compute high-quality alignments of a set of orthologous promoter regions from different vertebrate species. Unlike previous citizen science applications, Phylo intentionally hides much of the science behind it. A central idea of our contribution is to reduce the human computing part to a casual game, a puzzle, in order to broaden the spectrum of participants and collect the computing power generated by regular, non-scientist gamers. This approach expands to natural sciences the concepts of re-usability previously introduced by Luis von Ahn and co-workers in the ESP game [29] and reCAPTCHA [30]. Here, we apply our techniques to improve sections of a whole-genome MSA of 44 vertebrate species [31], produced by a state-of-the-art computer program called Multiz [16] and computed and made available by the UCSC Genome Browser group [32], which is used as the basis for comparative genomics studies by hundreds of researchers worldwide. We extract regions of the MSA having a low confidence score (i.e. regions that are likely to be misaligned) and we convert them into puzzles. These puzzles are made accessible on the web through a flash or javascript game interface, where web users can play them. Solutions are automatically sent back to our server, evaluated, and re-inserted in the original alignment in order to produce a higher quality MSA.

The paper is organized as follows. In the Results section, we describe Phylo and the set of alignments considered and show evidence of the effectiveness of our approach at improving alignments. In the Methods section, we detail the game mechanism and explain how the data are validated and re-inserted into the original MSA. Finally, we conclude by discussing the perspectives offered by citizen science approaches.

## Results

### Game overview

**Figure 1:**
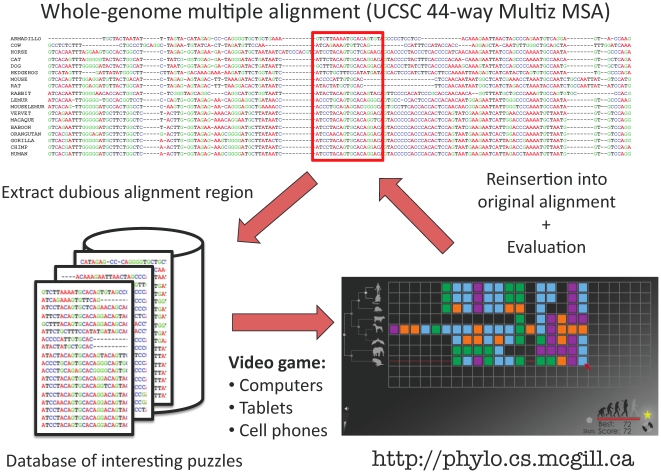
Phylo crowd-sourcing system for local improvement of multiple genome alignments.

Phylo is a citizen computing framework for local improvement of multiple sequence alignment. Figure 1 provides an overview of the system. Starting from a large multiple sequence alignment (in our case a 44-vertebrates whole-genome alignment), we focus on the promoters of genes known associated to be associated to specific diseases (see the “Data selection” subsection), identify short alignment regions that show signs of misalignment, and build a database of these subalignments. When a player starts a game, one of these subalignments is selected based on criteria provided by the player (or at random), turned into a puzzle-like game, and sent to him/her. Upon completion of the puzzle, the player's solution is sent back to our server. The solution is reinserted into the global alignment and evaluated. If it is better than the original alignment, it is retained.

Phylo aims to convert a MSA problem into a puzzle game that can be easily understood and played by web users through a flash or javascript interface (Figure 1). Here, DNA sequences are represented by strings of blocks of four different colors representing the four different nucleotides of the genetic code. We display these strings inside a matrix of up to 24 columns and 8 rows, where each row corresponds to one sequence. Each block can be moved horizontally, if necessary pushing its neighbors, but cannot be swapped with another block. As in any alignment, the goal of Phylo is to move the blocks in order to find a configuration that maximizes conservation across columns while minimizing the number of gaps. The game also displays a phylogenetic tree for the set of sequences considered, with each species being represented by an avatar.

We score the puzzles using a simple but realistic, easy-to-understand maximum parsimony algorithm that predicts ancestral sequences from the given alignment and sums the scores of the induced pairwise alignments, over all branches of the tree (see Methods). The pairwise alignment scoring scheme is a simplified version of that used in Blastz [33]. As the player modifies the alignment, the score is automatically recomputed and displayed.

Several mechanisms have been added to increase the entertaining value of the game while helping players achieve good solutions. First, the sequences are progressively added. The game starts with two sequences and the player must find an alignment with a score that is at least as good as the score of the original alignment (i.e. the alignment that has been pre-calculated by Multiz). We call the score of this alignment the “par” to be allowed to proceed to the next stage. Then, another sequence is added to the puzzle and the process is iterated until all sequences have been added. Note that contrary to the classical progressive alignment approach, players are allowed to revise any part of the alignment at any point. The second feature we added is a timer. Each stage must be completed within a certain time limit. In addition, we have also implemented a ranking system that records the number of puzzles solved by each registered user, and displays the list of the top 20 contributors. Together, these features aim to stimulate the competitiveness between players. Finally, we have implemented multiple mechanisms of puzzle selection. Players can either choose a puzzle by its difficulty level or by the type of disease the corresponding gene is associated to.

### Data selection

To evaluate the effectiveness of crowd computing at multiple sequence alignment, we selected a set of human promoters associated to genes with known implications in various diseases from the OMIM database [34]. OMIM diseases were assigned to one of seven broad disease categories (“Blood and immune system”, “Brain and nervous system”, “Cancer”, “Digestive system”, “Heart and circulatory system”, “Metabolic disorders” and “Sensory system”) based on an automated keyword matching procedure. The three largest categories are “Brain and nervous system”, “Cancer” and “Metabolic disorders”, which are each accounting for approximatively 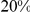 of the puzzles. Phylo players can choose puzzles based on the disease category, which gives the player a better feeling of (indirectly) contributing to biomedical research.

For each selected promoter (1 kb region upstream of the annotated transcription start site), we then extracted the corresponding sections of a 44-species multiple genome alignment [31] produced by the Multiz program [16] and available through the UCSC genome browser [32]. This multiple alignment comes in the form of a set of alignment blocks, where each block (ranging in size from a handful to several hundred columns) contains presumably orthologous DNA regions from some or all of the 44 species considered. Each selected alignment block was then scanned, using simple criteria described in Methods, to identify 24-column regions that (i) were most likely to contain alignment errors and (ii) were suitable to make interesting and challenging Phylo puzzles. When the alignment block contains more than 8 species, a subset is selected, aiming to maximize the phylogenetic diversity, to form the puzzle sent to the player (see Methods). A set of 739 puzzles were thus created.

### Game statistics

**Figure 2:**
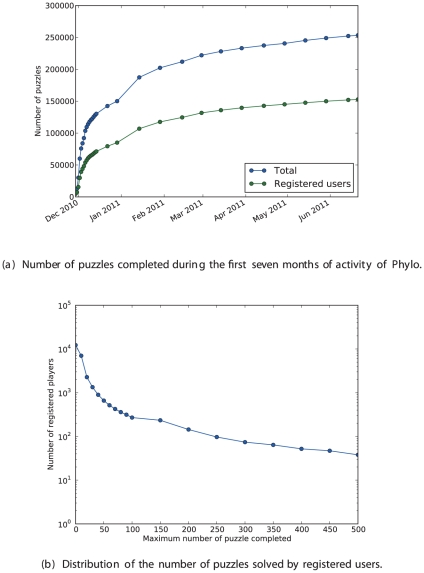
Statistics on the number of players. The top figure shows the number of puzzles played by registered and anonymous players during the seven first months of Phylo. The bottom figure shows the number of registered players w.r.t. the number of puzzle they solved.

Phylo was officially released on November 29, 2010 [35]. Here, we analyze and discuss the usage and performance statistics collected over the first seven months of activity. To date, Phylo counts 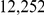 registered players, including 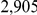 *regular players*, who logged in multiple times. Figure 2(a) shows the number of games completed by registered and non-registered players since the game release. As anticipated, due to the novelty of the game and thanks to broad media coverage, we had a larger number of new participants during the first month of activity. The number of games played daily stabilized since January 2011 and now we collect about 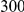 puzzle solutions per day. Users played a total 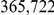 puzzles and reached the final stage 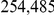 times (i.e. these puzzles are said to be *completed*; they are the only ones whose solution get transmitted back to our server). Roughly two thirds of the puzzles are solved by registered members.

Phylo is not equally attractive to all players. Registered users completed an average of 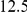 puzzles, but this number increases to 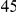 for regular users. In Figure 2(b), we detail the number of puzzles completed by registered users. Not everyone likes playing Phylo: 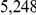 (42%) of registered players failed to complete a single puzzle. Although 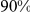 of the registered players completed less than 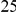 puzzles, the 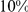 most prolific ones contributed almost 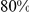 of the 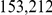 solutions returned by registered users. All top 20 contributors have played more than 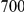 puzzles and the top player (username “stephano”) completed more that 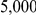
! Finally, we observe that 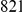 different registered users obtained the best score recorded for at least one puzzle, suggesting that even occasional players with little training can successfully contribute.

**Figure 3:**
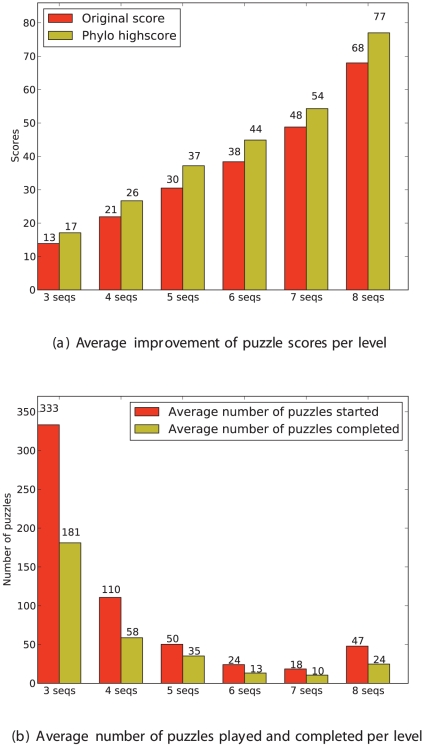
Statistics on the performance of players as a function of the number of sequence in the puzzle. (a) Average Phylo score of original alignments (red) and average best score obtained (yellow). (b) Success rate per level: Average number of times a puzzle has been played (red), and average number of times a player reaches the final stage of a puzzle (yellow).

In Figure 3, we analyze the influence of the level of the puzzle, defined as the number of sequences to align, on the participation and success rate. Figure 3(a) compares the average scores of the original alignment (i.e. as extracted from the original MSA) and of the best score submitted to Phylo, as a function of the level. We observe that the improvement ratio is fairly constant and independent of the difficulty of the puzzle. Figure 3(b) shows the number of times users start and complete a puzzle (i.e. succeed to reach the final stage). First, we note that the easiest puzzles are much more often played than the more difficult ones. This was expected as all new participants use the entry levels to practice and get more familiar with the game rules. Interestingly, at the other end of this scale, the trend is reversed and the participation increases for the higher level (i.e. MSA's with eight sequences). This observation suggests that, as intended, experienced players are more interested in solving the most challenging puzzles. Once again, the success rate seems independent of the difficulty and is roughly equal to one-half. This appears to us as a good balance between the accessibility and the competitiveness of our game.

### Alignment improvements

Puzzle solutions with score better than the par were sent back to our database. Each solution was re-inserted into the original alignment block and sequences that had been left out from the puzzle were re-inserted into the alignment (see Methods). Alignments were scored by inferring the corresponding ancestral sequences using a maximum likelihood approach and summing the pairwise alignment scores over all branches of the phylogenetic tree (see Methods). Four types of alignments strategies were evaluated:

1. **Original Multiz alignment** is the MSA produced by the Multiz program, without any realignment.
2. **Phylo-based alignment** is the MSA obtained by reinserting a solution to the Phylo puzzle into the original Multiz alignment, and completing it by adding the species that had been left out of the puzzle.
3. **De novo alignment** is the MSA produced by applying the alignment completion algorithm as in (2), but starting from an empty alignment instead of starting from the Phylo puzzle solution.
4. **Multiz-completed alignment** is the MSA obtained by applying the alignment completion algorithm to a version of the original Multiz alignment where only the set of sequences present in the corresponding Phylo puzzle are retained.

For each alignment block, the Phylo-based alignment was built from each of the different puzzle solutions, irrespective of the Phylo score they obtained. Each completed alignment was scored and the highest-scoring alignment was retained. Original and improved alignment blocks are available in Supplementary Material. Overall, the best Phylo-based alignment outscored the original Multiz alignments for 70% of the alignment blocks. In fact, even the score of *average* Phylo-based alignment exceeded that of the Multiz alignment about half the time. To rule out the possibility that alignment improvements may be only due to the alignment completion algorithm rather than the Phylo puzzle solutions themselves, we also compared the scores of the Phylo-based alignments to those of the de novo realignment and the Multiz-completed realignment. Phylo-based alignments produce strictly better scores than the three other approaches for 36% of the puzzles, while the de novo alignment outperformed the other three in 46% of cases. Original Multiz and Multiz-completed alignments outperform the other three for only 9% and 8% of the puzzles respectively. Taken together, these results suggest that improved alignments result from a combination of a better multiple re-aligner and a set of high quality initial solutions produced by Phylo players. Although the magnitude of the alignment score improvement is generally small (relative score increase of less 10% for 78% of the alignments), these improvements are important for a number of applications that heavily rely on alignment accuracy, including phylogenetic inference, identification of sites under selection, or RNA secondary structure prediction.

Recall that the puzzle score shown to the user only measures the quality of the solution to the alignment puzzle itself, outside of its alignment block context and with only a subset of the species present in the full alignment block. An interesting question is whether this score correlates with the final score of the alignment after its completion and reinsertion into the full alignment block. This correlation is weak, with only 55% of the puzzles played at least 5 times showing a positive correlation between Phylo score and final alignment score. Note however that puzzle solutions are only returned to our server if they achieve a score at least as good as the “par”, which means that only “good” solutions are considered. This suggests that the Phylo puzzle solutions form a good pool of initial solutions based on which improved multiple alignments can be obtained, but that the Phylo scores themselves (or at least those beating the par) are not very indicative of the quality of the alignment when placed in its context and extended to the full set of sequences. In that case, puzzles played a large number of times would have a better chance of producing improved alignments. Indeed, this is the case: 77% of the puzzles with at least 5 different Phylo solutions yield an improvement over the original alignment, whereas this fraction drops to 53% for puzzles with at most two different solutions.

Unsurprisingly, the number of species in the puzzle has an impact on the quality of the completed alignments that can be obtained from them. Small puzzles (3 or 4 sequences) result in improved alignments less than 63% of the time, while this percentage goes up to 73% for larger ones (size 7 and above). This is despite the fact that small puzzles are played on significantly more often than larger ones (2-fold difference in number of different solutions).

## Discussion

In this paper, we showed that a citizen science approach can be applied to improve the accuracy of multiple sequence alignments. More importantly, we demonstrated that we can turn this problem into a intuitive and entertaining computer game suited for casual gamers without any scientific background. Contrary to existing alignment editing tools such as JalView [40] and Seaview [41], which are designed for biology or bioinformatics experts, Phylo turns alignment optimization into a casual game. Unlike other citizen science projects such as Foldit, Phylo intentionally decouples the scientific problem from the game itself, such that even non-expert users can produce valuable solutions without significant scientific training. Instead of proposing to users an immersion into a theoretical scientific universe, we offer to web users a casual tetris-like video game to entertain themselves with the knowledge that their effort will be recycled to improve the analysis of biological data. We implemented this methodology and released our application on November 29th 2010. In 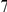 months, our server collected more than 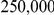 solutions generated by a community of more than 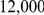 registered users. To these players, we must add all other players who played anonymously. These numbers demonstrate the impact on society of an approach combining casual computer games to citizen science projects.

In this work, we applied our methodology on a 44-way Multiz MSA from the UCSC genome browser [32] and use the solutions generated by the players to improve the MSA of 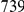 promoter regions. Our results are now publicly available at http://phylo.cs.mcgill.ca. This demonstrates that crowd sourcing yields practical improvements to the accuracy of MSA's. In future work, we plan to expand the range of application of “casual” citizen science techniques to other bioinformatics problems such as the RNA sequence/structure alignment and phylogenetic inference.

The clarity and the simplicity of the design that characterizes Phylo is an important asset to ensure the popularity of our game. In particular, we abstract the nucleotides to coloured blocks and develop an intuitive yet realistic scoring scheme that is well supported visually by various aspects of the game interface. This allows players to solve puzzles MSA's with up to 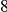 sequences – a problem size beyond the capacity of exact MSA algorithms [37]. In future work, we will continue to improve our game design in order to increase the size of the puzzles while maintaining the playability of the game. We will also include more features such as the addition of flanking columns representing the context from which the MSA fragment was been extracted, essential for players to correctly gauge how end gaps should be penalized. This mechanism should enable us to increase the correlation between the Phylo puzzle scores and the final alignment scores after reinsertion of the puzzle solutions into their original alignment blocks.

An interesting related question is how best to harness crowd computing for improving alignments: one wants the player community to work on as many regions of the alignment as possible, but also to do as good a job as possible at improving each of them. As discussed previously, the more often a puzzle is played on, the better the chances of producing good alignments. However, the value of additional solutions diminishes as the number of available solutions increases. While our current dispatching system assigns puzzles to players in a random manner (subject to an user's preferences about problem size and disease associations), a better approach would be an adaptive approach where we monitor, for each puzzle, the number of different solutions obtained to date and the number of people who played on it. Puzzles whose solution space seems to saturate (i.e. the same solutions are found over and over again by the players) should be considered solved and stop being fed to players. Similarly, puzzles that are rarely completed by the players may have properties that makes them boring or too challenging and should stop being sent to users. Adapting puzzle dispatching may even go further and detect a specific player's preferences or skills via the set of solutions produced to date and select new puzzles on that basis.

Finally, we conclude this paper by discussing the validity and the scientific impact of citizen science frameworks. Above everything, the question of the computational tractability of the problem addressed is fundamental. Indeed, to be scientifically justified, this strategy must demonstrate that human expertise is necessary and that computer programs cannot perform better. We believe that any citizen science approach applied to well-defined scientific problems must satisfy these three criteria: (i) Computational difficulty of the problem, (ii) range of application of exact methods, and (iii) comparison with heuristic methods. Here, we stress that the MSA problem using a maximum parsimony score has been shown 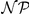-hard [18], [42]. Moreover, it has also been shown that exact methods cannot be applied on MSA's with sizes similar to those used in Phylo [37]. However, the question of whether an algorithm using heuristics can outperform humans remains. To address this point, we showed in this paper that the alignments produced by using Phylo puzzle solutions as basis generally improve on both the genome-wide alignments produced by Multiz as well as a custom multiple alignment heuristics that specifically aims at optimizing the objective function used in this study.

However, computational considerations are not the only ones of interest. Another fundamental aspect of this game is its role toward educating people to the challenges encountered in computational biology and discrete optimization in general, as well hinting to some important evolutionary biology and genetic concepts. Although Phylo intentionally abstracts the scientific context of MSA's to an intuitive casual game, it also offers a portal for anyone looking for information on the subject. More precisely, two games menu sections titled “About” and “FAQ” describe the biological motivations, the scoring scoring algorithm and how the puzzle solution are used. Moreover, over the last year, our interface has been already used in several classes and public demonstrations around the world to illustrate genomic research.

Billions of “human-brain peta-flops” of computation are wasted daily playing games that do not contribute to advancing knowledge. While only a small fraction of important computational problems are amenable to crowd computing, and translating those that are into fun, intuitive games can be challenging, the reward of a well-designed framework for human computation, combined with a wide user-base, is access to a huge basin of computing power.

## Methods

### Puzzle scoring scheme

The interface of Phylo displays a simplified and entertaining representation of an MSA instance with its associated phylogenetic tree. Each nucleotide is represented by a block whose colour corresponds to its type (i.e. Adenine, Cytosine, Guanine and Thymine). The scoring scheme for a given puzzle alignment must be evolutionarily realistic while being intuitive and fast to compute (as it is recomputed in real time every time the player modifies the alignment). To evaluate a given alignment, the game starts by inferring ancestral nucleotides or gaps at each ancestral node of the phylogenetic tree using a maximum parsimony approach (Fitch algorithm [36]), considering a gap as a fifth character, independently for each position. It then sums, over all edges of the tree, the score of induced pairwise alignments, each evaluated using an affine gap cost model. In order to make the scoring intuitive, our scheme uses integer values (Match score = 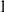, mismatch score = 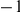, gap opening score = 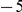, gap extension = 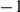) that approximate those used by Blastz [33]. We note that because it infers ancestral nucleotides independently at each position, the original Fitch algorithm is not designed to accommodate an affine gap penalty model and may result in suboptimal ancestral sequences, which would yield a pessimistic alignment evalution. However, exact algorithms or better approximations are computationally more expensive [18], [37], and we considered that the simplicity of our scoring method and its speed largely compensate for the slight accuracy loss.

### Puzzle database construction

Our puzzle database is based on a multiple sequence alignment of 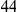 vertebrate species available on the UCSC genome browser [32]. Human genes associated to diseases were first downloaded from OMIM [34] and the alignment regions corresponding to their promoters (1 kb region upstream of annotated TSS in human) were extracted from the whole-genome alignment. Each promoter's alignment (which can consist of several alignment blocks) is then scanned to identify blocks that are possibly misaligned and that may produce interesting and challenging puzzles, according to the following criteria. An alignment block is said to be “interesting” if its ratio 
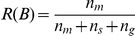
 of matches (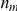) versus mismatches (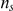) and gaps (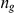) is at most 0.55, ensuring that it includes many gaps and mismatches and thus has a high probability of being non-optimal.

Interesting blocks are typically longer and contain more species than our game can accommodate. The number of species in the alignment is first reduced to at most 8, by keeping the first 8 species according to our species ranking list, which aims at selecting a set of species as phylogenetically diverse as possible (i.e. whenever possible, select distantly related species). We then scan each reduced “interesting” block with a sliding window of size 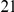 and select a frame position if the corresponding sub-block 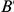 has a good number of mismatches and gaps (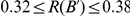) and a good degree of sequence length variability: 
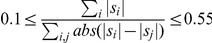
. All threshold values have been determined empirically to produce challenging puzzles. Finally, selected puzzles are given a unique identified and stored in a MySQL database from which the game interface retrieves puzzles to give to players.

### Alignment completion algorithm

Recall that a Phylo puzzle consists of a slice of 24 columns taken from the original UCSC 44-way multiple alignment, and then reduced to a set of at most 8 species. To be useful, a solution to the puzzle must be reinserted into the original alignment block and completed by adding any left-out species to the alignment. This is performed as follows. Consider an alignment block 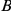 with a set of species 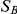, from which was extracted a puzzle with solution 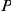 over the set of species 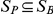. We first consider the subalignment 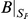 formed by the species contained in 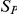, and replace the region corresponding to the puzzle by 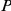, to obtain a revised alignment 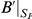. We then use the following algorithm to add to 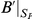 the set of sequences from species in 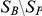. Sequences are added one by one. When adding sequence 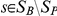 to the current alignment, we start by first inferring the ancestral sequence probabilistic profile of the immediate ancestor of 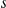 (based on the current alignment), using the Ancestors 1.0 program [38]. We then align 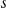 to that profile so as to maximize the expected alignment score, using a dynamic programming algorithm similar to the Needleman-Wunch algorithm [39]. Aligning 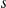 to its ancestral profile implicitly defines how to add it to the current alignment. Once added, the process continues with the next sequence to be added, until all sequences have been reinserted. Note that the process can also be started from an empty alignment (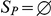), in which case this can be considered as a fully automated realignment algorithm refered to as “de Novo realignment” in Results. It can also be started from 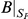, which results in an alignment we call “Multiz-completed alignment”.

### Alignment block evaluation

For the purposes of comparing the accuracy of the various alignment strategies proposed, maximum likelihood ancestral sequences for a given alignment block are first inferred using Ancestors 1.0 program [38]. Then, for each branch of the phylogenetic tree, we calculate the score of the pairwise alignment induced by the MSA and the ancestral reconstruction, using the Blastz substitution and affine gap scoring schemes [33]. The final score of the alignment is the sum, over all branch of the tree, of the pairwise alignment scores.

### Implementation

The original client interface has been implemented in a Adobe Flash Actionscript 3.0. More recently, we released a javascript interface to improve the portability of our system and enable users to play Phylo on tablets and other mobile devices. The server has been implemented in Java. The client connects to the server via XMLSocket and the server listens through SocketServer. The communication between the server and the MySQL database is supported by JDBC drivers. Finally, password of registered users are encrypted SHA-512 and Salting to ensure user privacy.

### Availability

Phylo is open, free of charges, to academic users who are willing to use it to improve their MSA data. Interested users are invited to send us data using the MAF format at phylo-submit@cs.mcgill.ca. Sequences should be pre-aligned using a computer program, preferably together with a confidence score. Once submitted, the data will be scanned in order to create new puzzles. Once every puzzle will have been played a predefined number of times (by default 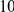), the solutions will be re-inserted in the original MSA and the results will be sent back to the user. All information about the submission process are available at http://phylo.cs.mcgill.ca/eng/submit.html.
